# Integration and benefits of root inoculation with endophytic entomopathogenic fungus *Metarhizium brunneum* in the propagation of olive tree seedlings in nurseries

**DOI:** 10.1128/aem.02048-25

**Published:** 2026-01-30

**Authors:** A. Romero-Conde, M. Yousef-Yousef, P. Valverde-García, A. Sandoval-Lozano, E. Quesada-Moraga, I. Garrido-Jurado

**Affiliations:** 1 Departamento de Agronomía, Unidad de Excelencia María de Maeztu DAUCO, ETSIAM, Universidad de Córdoba 16735, Córdoba, Spain; Royal Botanic Gardens Kew, Surrey, United Kingdom

**Keywords:** insect pathogenic fungi, growth promotion, microsclerotia, endophytism, arbuscular mycorrhizal fungi, induced systemic resistance

## Abstract

Despite the recognized benefits of endophytic entomopathogenic fungi (EEPF) as biological control agents, their potential role in promoting plant growth and inducing systemic resistance (ISR) in olive nurseries remains poorly understood. Many fungi live in close association with plants, and a deeper understanding of the interactions among beneficial fungi is required to unravel the complex relationships among olive cultivars and phytopathogens, which can be fundamental for improving crop production and protection. EEPF are important soil-inhabiting microorganisms that can provide several benefits to olive plants beyond insect pest control. This study investigates the role of *Metarhizium brunneum* in the olive rhizosphere during the nursery stage, focusing on its potential to promote plant growth and induce systemic resistance in Picual and Manzanilla cultivars. Conidia and microsclerotia of the *M. brunneum* EAMa 01/58-Su strain, together with an arbuscular mycorrhizal fungus (AMF), were applied to olive plants. Fungal colonization, growth promotion, and induction of systemic resistance were evaluated. Microbiological, qPCR, and droplet digital PCR analyses showed that *M. brunneum* colonized roots via the apoplastic pathway when applied as conidia or microsclerotia. Mycorrhization rates were higher in Manzanilla, but elevated mycorrhization inhibited *M. brunneum* colonization. Growth-promoting effects were more pronounced in Picual. Application of the EAMa 01/58-Su strain induced specific subsets of systemic resistance (SR) genes associated with the ISR pathway. Additionally, Picual plants treated with both AMF and microsclerotia exhibited increased leaf nitrogen and potassium levels. Overall, this strain demonstrates promising potential as a plant growth promoter during the early nursery stage of olive cultivation.

**IMPORTANCE:**

Olive cultivation is an economically and culturally significant crop; however, early-stage plant growth in nurseries can be limited by nutrient uptake, abiotic stress, and pathogen pressure. Endophytic entomopathogenic fungi (EEPF), such as *Metarhizium brunneum*, are traditionally used for biological control of insect pests, but their potential to promote plant growth and induce systemic resistance has been overlooked and is currently of interest to olive nursery companies. This study demonstrates that *M. brunneum* EAMa 01/58-Su can effectively colonize olive roots, improve certain vegetative growth parameters, and activate systemic resistance pathways, influenced by both cultivar and the application with arbuscular mycorrhizal fungi prior to field transplantation. These findings highlight the dual role of the *M. brunneum* EAMa 01/58-Su strain in olive nurseries, providing both pest protection and growth-promoting benefits. Integrating EEPF into nursery management could enhance plant health, nutrient status, and stress resilience, offering a sustainable strategy for early-stage cultivation and contributing to more productive and resilient olive orchards.

## INTRODUCTION

The endophytic entomopathogenic fungus (EEPF) *Metarhizium* spp. (Ascomycota: Hypocreales) is naturally present both below ground (soil and rhizosphere) and above ground (phylloplane and endophyte) in different ecosystems in temperate, subtropical, and tropical regions in natural or cultivated habitats such as: holm oak dehesa, holm oak reforestation, organic olive orchard, conventional olive orchard, citrus orchard, sunflower, and wheat plantations (1–8) and plays an important role in the control of various pests in different crops, such as corn, wheat, rice, and olive cultivation (9–12). Its main reservoir is the soil, which generally offers the greatest potential for pest control, as it is estimated that 80% of insect pests dwell or spend part of their life in the soil (13). *Metarhizium* spp. behavior in the soil is influenced by several factors that affect its persistence, effectiveness, and ability to interact with other organisms (14). *Metarhizium* spp. can be applied for pest control as conidia or as the resistance structure, microsclerotia (MS), which allow it to survive for long periods under adverse conditions of temperature, moisture, and ultraviolet radiation (UV-B) mainly (15). Recently, there has been great interest in this structure within the plant protection sector, mainly due to its ease of mass production (9). Besides, nursery companies have demonstrated increasing interest in the use of EEPF, particularly through the use of propagules such as MS, owing to the advantages they offer over conidia (16).

*Metarhizium* spp. is naturally present in the bulk soil, as previously mentioned, which facilitates its establishment in the plant rhizosphere (7, 17, 18). Furthermore, it can colonize the rhizosphere and establish as an endophyte within plant tissues (19). This capability allows it to closely interact with the three components of the rhizosphere (the rhizospheric soil, the rhizoplane, and the root), thereby benefiting plant development (20, 21). The rhizospheric effect may be facilitated by the release of substances from the root borders of the plants, which could even be influenced by the plant cultivar (22) and promote the development of an active microbial population, or it may positively interact with other soil microorganisms (bacteria, fungi, etc.), contributing to a more balanced microbiome that gives rise to a complex web of microbial inter- and intra-kingdom interactions relate to plant health to suppress diseases in the soil for use in agriculture (23). Additionally, within the rhizosphere, it can enhance the availability of essential nutrients for the plant (i.e. nitrogen, phosphorus, and iron) (24–26), stimulate hormone production and increase tolerance to abiotic and biotic stresses, and therefore promote plant growth (27–32). Moreover, the activation of the plant’s immune system, protecting it from pests and diseases through the induction of systemic resistance (SR) via the stimulation of ethylene (ET), jasmonic acid (JA), and salicylic acid (SA) can occur as a result of these interactions (33–36).

The strain EAMa 01/58-Su of the EEPF *M. brunneum* Petch. represents a promising biological control tool when applied to the soil and rhizosphere of various crops, and it has shown great multifunctionality in controlling insect pests triggered defensive system induction in the antibiosis and compensatory growth to protect plants from pest damage (36) and could even be compatible with mycorrhizal inoculation in olive plants in the nursery. In addition, it improves the bioavailability of this nutrient in calcareous and non-calcareous soils for sorghum and sunflower plants (37).

To date, *Metarhizium* spp. has not been found, either naturally or artificially, to be competent in the rhizosphere or as an endophyte in the olive tree (38).

The propagation and multiplication of olive trees in nurseries are commonly performed through the rooting of semi-hardwood cuttings under misting conditions within greenhouses managed under controlled environments (39). Prior to transplanting to the field, the rooted seedlings are transferred to nursery conditions for commercialization and are typically inoculated with arbuscular mycorrhizal fungi (AMF), which are obligate symbionts that enhance root establishment by improving nutrients and water uptake from the early stages of cultivation, and which AMF may also increase stress tolerance after field transplantation (40). However, to date, no studies in olive trees have evaluated the interaction between AMF and EEPF such as *M. brunneum,* when co-applied to the soil.

The use of AMF in olive propagation has mainly focused on the transplanting of cuttings into pots, improving nutrient and water uptake, soil aggregation, pathogen suppression, and tolerance to abiotic stress. However, little is known about how EEPF interact in the olive rhizosphere. Recent studies in other crops have shown that AMF and EEPF co-inoculation can have synergistic or antagonistic effects, depending on the fungal species and the host (41, 42). Their combined effects have not been evaluated in perennial woody plants, nor has the compatibility of different EEPF and AMF species been fully explored. In olive, these interactions remain largely unknown, representing a promising area for research in sustainable nursery management.

Consequently, the present study aims to elucidate the role of conidia and MS produced by the strain EAMa 01/58-Su from *M. brunneum* in the olive rhizosphere by evaluating: (i) the colonization of the olive rhizosphere; (ii) the plant growth promotion; (iii) the compatibility of inoculating nursery plants with EEPF together with an AMF; and (iv) the induction of systemic resistance through the activation of ET, JA, and SA, that can prepare the plant for future biotic or abiotic stresses.

## MATERIALS AND METHODS

### Fungal inoculum used in the experiments

The strain EAMa 01/58-Su of the EEPF *M. brunneum* was obtained from the culture collection of AGR 163 Research Group “Agricultural Entomology” from the Department of Agronomy of the University of Cordoba. The strain was isolated from a wheat field located in Hinojosa del Duque (Córdoba, Spain) and is deposited in the Spanish collection of culture types (CECT) with accession number CECT 20764. This strain was exclusively licensed by the University of Cordoba to the company KOPPERT B.V. in 2021, and it was selected in this work for its ability to produce conidia and MS (15).

Commercial inoculum of Myco-Soil (Agrogenia, Cordoba, Spain) consisting of 300 propagules per gram of *Rhizophagus intraradices* (NC Schenck y GS Sm.) C. Walker y A. Schübler (previously *Glomus intrarradices*) (Mucoromycota: Glomerales) was used to provide mycorrhization. These AMF were included in the experiments, because they have been frequently used in olive nurseries to produce new plants in recent years, and natural mycorrhization also usually occurs in the field (43).

Conidia production was obtained by scraping the mycelium from fresh actively growing cultures on malt-agar Petri plates (Biolife, Milan, Italy) in an aqueous solution of 0.1% Tween 80. Conidial suspensions (CS) were adjusted to 1 × 10^9^ conidia/mL using a Malassez chamber. MS production was carried out in our laboratory with controlled conditions following the protocol described by Yousef-Yousef et al. (15).

### Plant material, fungal treatments, and experimental design

The olive plants of two cultivars (Picual and Manzanilla) were produced by a commercial olive nursery in Encinarejo (Cordoba, Spain). These two cultivars were selected because of their international agronomic relevance: Picual is the most widely cultivated olive variety, accounting for nearly 20% of the world’s olive oil production due to its high yield, oil stability, and adaptability (44–46). In contrast, Manzanilla is the most widely appreciated table olive cultivar, recognized for its fruit quality, ease of processing, and protected geographical status (44, 46–48). Their contrasting agronomic traits and global significance make them suitable models to investigate cultivar-dependent interactions with beneficial fungi. The 20 cm plants were transplanted to new pots (8 L) with the Plantaflor substrate (Plantaflor Humus Verkaufs-GmbH, Oldenburg, Alemania) with a mixture of 35% white peat, 65% black peat, NPK (18-10-20) fertilizer, with an electrical conductivity (10–80 mS/m) and a pH (H_2_O): 3.5–7.0, as potting medium. The AMF were added to the pots following the manufacturer instructions only in the mycorrhized treatments. Plants were fertilized with Welgro Standard plus (Sipcam Jardín, S.L., Valencia, Spain) and also a multipurpose contact insecticide Cythrin Garden (Comercial Química Massó, S.A. Cinisello Balsamo, Italia) was applied. The plants were grown for 14 days until the beginning of the experiment to ensure that mycorrhization had occurred in the AMF treatments. The experiment was carried out in a greenhouse with controlled conditions, maintaining throughout the experimental period the mean daily relative humidity from 25.81% to 74.67% and the mean daily temperature was maintained from 12.76°C and 22.10°C (Fig. S1).

The treatments included were: control (C), AMF applied to the soil (AMF), soil drenching with CS, MS sprayed on the soil (MS), AMF applied to the soil simultaneously with soil drenching by conidial suspension (AMF + CS), AMF applied to the soil and soil drenching with conidial suspension thirty days later (AMF + CS30), AMF applied to the soil simultaneously with MS sprayed on the soil (AMF + MS) and AMF applied to the soil and MS sprayed on the soil thirty days later (AMF + MS30). In the conidial treatments (CS, AMF + CS, and AMF + CS30), 5 mL of conidial suspension was added to each pot, and 5 g of MS was added to each pot in the MS treatment (MS, AMF + MS, and AMF + MS30). Each treatment comprised a total of 10 plants. Growth parameters measurements were taken every 30 days. Of the 10 plants per treatment, 3 plants were removed after 60 days to measure fungal colonization using microbiological and molecular techniques and to evaluate systemic resistance genes in the plant. At the end of the experiment (120 days), all parameters evaluated were measured: mycorrhization rate, persistence of *Metarhizium* in the soil, fungal colonization, growth parameters, expression of resistance genes in the plant, content and presence of nutrients in the leaf, and histopathology of olive roots treated with *M. brunneum*. The experiment was carried out under shade house conditions, and temperature and HR were registered with a Gemini Data Loggers (Tinytag Plus Dual Channel, Chichester, UK). The experiment lasted for 120 days, at which point the olive trees can be marketed as seedlings, provided they have reached sufficient age and development to survive transplantation in the field.

### Data collection of vegetative growth

The effect of the experimental treatments on vegetative growth was assessed at monthly intervals over 120 days. The following measurements were recorded: height of the primary axis (cm), length of two selected shoots (cm), stem diameter (cm), number of shoots, and the chlorophyll content with a handheld chlorophyll meter (Soil Plant Analysis Development: SPAD-502) (Minolta Corporation, Ltd, Osaka, Japan). At the end of the assay, at 120 days, the plants were uprooted, and biomass (fresh and dry matter weight) was evaluated. Fresh weights were recorded for each plant. Then, the fresh samples were dehydrated at 60°C for 14 days (until reaching a constant weight), which was considered the dry weight. Finally, leaf nutrient analysis was performed by CSR Laboratorios (Jaén, Spain) with 250 leaves sampled per plant.

### Mycorrhization rate in olive roots

Mycorrhization rate was evaluated at the end of the experiment. Plants were harvested and representative samples were collected from the entire root system. Specifically, fine roots with a diameter ≤1 mm were selected, as lignified roots of larger diameter do not support mycorrhizal symbiosis, as the company indicated. For each plant, 5 g of recently developed roots were collected. The roots were gently shaken to remove excess substrate but were neither washed nor dried to preserve their natural state. Samples were placed in sealed plastic bags to maintain their intrinsic moisture and stored at 4°C until delivery to Intra Radice S.L. (Granada, Spain). Mycorrhization rate was calculated as the mean of three technical replicates per sample.

### Persistence of fungal inoculum in soil

Soil samples were collected at the beginning and end of the experiment (120 days), and colony-forming units (CFU) per gram of soil were determined to assess the conidial density in each fungal treatment. One gram of soil was added to 9 mL of sterile distilled water and shaken for 4 h. After homogenization, aliquots of 100 μL were spread onto Sabouraud Glucose Agar Chloramphenicol (SGAC) medium in Petri dishes and incubated at 25°C for 7–10 days (49).

### Fungal re-isolation from olive tissues

Fungal presence in olive plant tissues was monitored at two time points, 60 and 120 days (end of experiment). For that, fresh adult leaves were taken at three different levels, while new roots with a diameter of 1mm were randomly selected from the root system. The leaf and root samples were immersed in 1% (wt/vol) sodium hypochlorite solution (PanReac AppliChem, Barcelona, Spain) for 1 min and rinsed two times in sterile distilled water. To ensure total disinfection of the leaves, 100 μL of the last rinse from each sample was cultured on SGAC plates. Consequently, after surface disinfection, the leaves and roots were air-dried in a laminar flow hood. The three leaves in each sampling were split into 5 fragments of 1 cm^2^ each, and the roots were split into 10 fragments that were 0.5 cm long. The fragments were plated onto Petri dishes containing SGAC medium, sealed with parafilm, and incubated in the dark at 25°C for 10 days. The proportion of fragments exhibiting fungal outgrowth was recorded.

### Detection and quantification of *Metarhizium brunneum* by quantitative PCR and droplet digital PCR (ddPCR) in olive roots

Fragments of disinfected roots for each treatment were collected in 1.5 cm Eppendorf tubes at 60 and 120 days. Their homogenization was made with a mixer mill grinding (Restch MM400, Düsseldorf, Germany). The DNA Extraction Kit Phytopure of Cytiva (Amersham Life Science, Buckinghamshire, UK) was used for plant DNA extraction. The DNA was quantified using a 2000c spectrophotometer (NanoDrop Technologies, Wilmington, USA). The primers used for quantification amplify the region of the nitrogen response regulator nrr (F) TCAGGCGATCTCGTGGTAAG and (R) GGGGTGTACTTGAGGAATGGG (50). The qPCR was performed in triplicate and was repeated two times. Each reaction consisted of 10 μL of 2× Real-Time PCR Master Mix (Sensifast, Meridian Bioscience & Fluorescein, OH, USA), 0.8 μL of each primer at 10 μM, 2 μL of DNA (45 ng in total), and 6.4 μL of PCR water to a final volume of 20 μL. The conditions were: 3 min at 95°C followed by 39 cycles of 95°C for 5 s, 60°C for 30 s, 1 min at 95°C, and finally from 65°C to 95°C with a transition range of 0.5°C every 5 s. The nrr gene was selected as a target for quantification because it is a well-conserved gene among nitrogen-fixing microorganisms and plays a key role in regulating nitrogen metabolism. Its expression is indicative of the presence and activity of nitrogen-responsive microorganisms in plant roots, making it a reliable molecular marker for assessing microbial colonization and function during the early stages of cultivation (50). In their study, primers targeting the *nrr* gene were utilized to detect and quantify the presence of *Metarhizium* spp. in the roots of *Phaseolus vulgaris*, demonstrating the utility of this gene as a molecular marker for monitoring fungal colonization in plant tissues. The qPCR reactions were performed using a CFX Connect (Bio-Rad). The threshold cycle (Ct) and standard curves were automatically generated by CFX Manager 1.1 (Bio-Rad), where Ct values were plotted against the logarithmic initial DNA concentration. The concentrations of the fungal genomic DNA standard curves ranged from 31 to 0.0031 ng, were enriched with 45 ng of plant DNA, and subjected to qPCR. The quantity of *M. brunneum* was expressed as a ratio of fungal DNA to plant DNA (µg g^−1^).

To determine whether it was possible to achieve a greater fungal detection in the samples that had tested negative in qPCR, we analyzed these samples using the ddPCR technique (51). For that, the ddPCR technique was optimized using fungal DNA (10 ng/µL) of *M. brunneum* EAMa 01/58-Su extracted from a pure culture, and Milli-Q water as negative control and the same primer used in qPCR. Temperature and concentration primer was tested to determine the optimal hybridization temperature ranging from 53°C to 63°C and primer concentration ranging from 50 to 300 nM. The optimal temperature was determined to be 61.5°C and the optimal primer concentration was 100–150 Nm for forward and 100–120 for reverse primer. The ddPCR reaction used per sample was of 2 μL of DNA, 11 μL of QX200 ddPCR EvaGreen Supermix, 2.2 μL of each primer at 200 Nm 4.6 µL PCR-grade water to a final volume of 22 µL. Of this volume, only 20 μL of mix per sample could be used than was run as single replicates with a volume of 20 μL of mix per sample. For each sampling mix, droplets were generated with the QX200 Droplet Generator (Bio-Rad) in cartridges containing 20 μL of the reaction mix and 70 μL of Droplet Generation Oil per well. Forty microliters of the emulsion volume was transferred from the cartridge to a 96-well PCR plate, and the PCRs were performed on the C1000 Touch Thermal Cycler (Bio-Rad). The thermocycling conditions were: 5 min at 95°C followed by 39 cycles of 95°C for 30 s, 61.5°C for 3 min, after that, 4°C for 5 min and finally a step of 90°C for 5 min for droplet stabilization. After amplification, the PCR plate was directly transferred to the QX200 Droplet Reader, screening each droplet for fluorescent signal.

### RNA isolation, cDNA synthesis, and reverse transcription-quantitative PCR (qRT-PCR) analysis

The genes selected for the study were designed by Pronacera Therapeutics SL (Seville, Spain) using the Kyoto Encyclopedia of Genes and Genomes (KEGG) database for each signaling pathway of interest for the species *Olea europaea* var. *sylvestris*. The primers designed for the chosen signaling pathways (ET, JA, and SA), as well as those for the ACT1 gene as a housekeeping gene, are listed in Table S1. Samples were taken from all treatments, consisting of one leaf from mid-height of each plant at 60 and 120 days. RNeasy Mini Kit (Qiagen, Germantown, MD, USA) was used to extract the total RNA from the leaf. The RNA was quantified using a 2000c spectrophotometer (NanoDrop Technologies). cDNA synthesis was performed using iScript gDNA clear cDNA synthesis kit (Bio-Rad, CA, USA) according to the manufacturer’s instructions. qRT-PCR reactions were performed containing 2 µL of cDNA, 0.5 µL of each primer (10mM), 10 µL of 2 × iQ SYBRH Green Supermix (Bio-Rad, Thermo-Fisher Scientific, Carlsbad, CA, USA) and sterile water for PCR up to a total volume of 20 µL. The following parameters were used in all reactions: 94°C for 5 min, 50 cycles of 94°C for 30 s, 55°C for 30 s, and 72°C for 40 s. Linear equations, correlation coefficients (*R*^2^), and reaction efficiencies were estimated for each transcript. Melting curves of qRT-PCR products were assessed from 55°C to 95°C to confirm the amplification of single PCR bands. For all samples, the reaction protocol was as follows: 5 min at 95°C for initial denaturation, cooling to 55°C and melting from 55°C to 95°C with a 0.5°C transition rate every 10 s (52). Each treatment had seven biological samples. Verification of amplification was performed by electrophoresis in 1% agarose gel. To visualize the gel, SYBR Safe DNA stain (Invitrogen, Thermo-Fisher Scientific, Carlsbad, CA, USA) was added. In the end, an image of the agarose gel was taken with a gel imaging system (BioRad Gel Doc EZ Imagen, Thermo-Fisher Scientific, Carlsbad, CA, USA). The relative expression of each gene was normalized to the ACT1 Ct value and calculated according to Livak and Schmittgen (53) following the 2^−ΔΔCT^ method. For all transcripts, reactions were repeated at least two times (independent qRT-PCR experiments), and each of these assays always included two biological replicas per transcript and plate.

### Histopathology of olive seedling treated with *M. brunneum* propagules

Fragments of olive roots treated with CS were fixed in 2% glutaraldehyde, postfixed in osmium tetroxide, dehydrated on an ascending scale of acetone, and then embedded in Epon 812 resin, polymerized in an oven. For optical examination, the blocks were sectioned with an ultramicrotome to a thickness of 400 nm (semi-fine), placed on a slide, and stained with toluidine blue on a hot plate. Finally, each sample was visualized with the Leica DM5000B optical microscope at the Central Service for Research Support (SCAI) of the University of Cordoba (Spain).

For transmission electron microscopy (TEM), the resin block was sectioned with an ultramicrotome to a thickness of 80 nm, placed on a Cu grid, and contrasted with uranyl acetate and lead citrate. The sample was visualized using a JEOL JEM 1400 transmission electron microscope at SCAI, University of Córdoba (Spain).

### Statistical analysis

Data related to the treatment effects over time on the following parameters: height of the primary axis, length of shoots, stem diameter, and SPAD were analyzed using a mixed model for repeated measures, with treatment, time, and treatment x time as fixed effects and treatment × replicate as a random effect. Treatment means were compared with the control using Dunnett’s test (*α* = 0.05). Analysis was performed with SAS PROC GLIMMIX (54).

Fungal colonization, mycorrhization rate, persistence of fungal inoculum in soil, biomass, induced resistance, and foliar nutritional analysis were analyzed using analysis of variance (ANOVA) with LSD test at *P* ≤ 0.05 for multiple comparisons (Statistix 9.0 statistical software, Tallahassee, FL, USA). Data are expressed as mean ± SE; *P* values less than 0.05 were considered statistically significant.

In ddPCR analysis, data were analyzed with QuantaSoft Software, version 1.7. A threshold was manually set up just above the amplitude value of the cloud considered as background. This threshold enabled the differentiation of droplets by categorizing them as positive (high level of fluorescence) and negative (low level of fluorescence). The number of copies of targeted DNA per μL was calculated with a Poisson model, comparing the number of positive and negative droplets out of a mean of 14,000 droplets. The PCR reactions that generated fewer than 10,000 droplets in total were excluded from the analysis, and a result was considered positive if at least three positive droplets were detected. To quantify the results of ddPCR in the same units as qPCR, a transfer of the number of copies of DNA/µL to ng of DNA/g of root was carried out.

Results of relative gene expressions were analyzed using one-way ANOVA followed by Dunnett’s test at *P < 0.05, **P < 0.01, and ***P < 0.001 to indicate significant differences from control (Statistix 9.0 statistical software). Data of gene expression represents the meaning of three or seven independent technical replicates, for 60 or 120 days, respectively.

## RESULTS

### Mycorrhization in olive roots

Natural mycorrhization was not detected in control treatments, nor in the treatments with the application of CS or MS. Mycorrhization was only detected in the treatments where AMF were added.

There were significant differences between the treatments in the mycorrhization rate within each olive cultivar, Picual (*F*_(4,10)_ = 5.63; *P* = 0.0123) but not in Manzanilla (*F*_(4,10)_ = 3.26; *P* = 0.0588). However, a higher percentage of mycorrhization in the Manzanilla cultivar than in the Picual cultivar was detected for all the AMF treatments.

**Fig 1:**
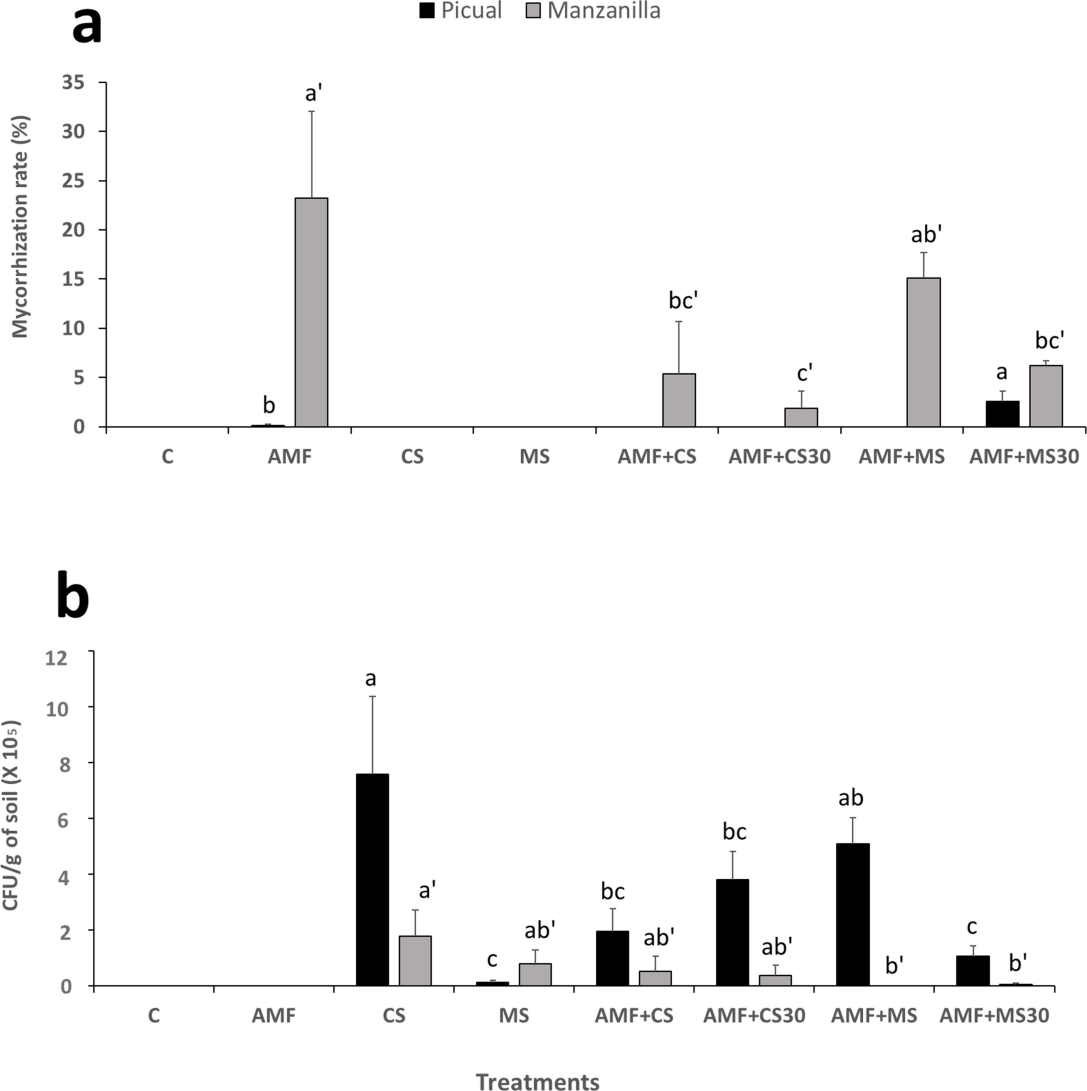
(**a**) Mycorrhization rate (%) detected in the olive cultivars Picual and Manzanilla 120 days after the treatments: mycorrhiza (AMF); mycorrhiza + conidia (AMF + CS); mycorrhiza + conidia applied 30 days after the application of mycorrhiza (AMF + CS30); mycorrhiza + microsclerotia (AMF + MS); and mycorrhiza + microsclerotia applied 30 days after the application of mycorrhiza (AMF + MS30). Values of mycorrhization rates within the same cultivar not sharing the same letter are significantly different at *P* ≤ 0.05 according to Fisher’s protected LSD test (*P* ≤ 0.05) (*n* = 5) (**b**) Persistence of *M. brunneum* EAMa 01/58-Su strain in the soil of both olive cultivars Picual and Manzanilla 120 days after the treatments: control (C); conidia (CS); microsclerotia (MS); mycorrhiza + conidia (AMF+CS); mycorrhiza + conidia applied 30 days after the application of mycorrhiza (AMF+CS30); mycorrhiza + microsclerotia (AMF+MS); mycorrhiza + microsclerotia applied 30 days after the application of mycorrhiza (AMF+MS30). Error bars represent the standard error (SE) of the mean. Values of CFU/g of soil within cultivar not sharing the same letter are significantly different at *P* ≤ 0.05 according to Fisher’s protected LSD test (*P*≤0.05) (*n*=6).

For the Picual cultivar, only the seedlings from the AMF and AMF + MS30 treatments showed mycorrhization, with 0.1% and 2.57%, respectively. Nonetheless, all treatments in the Manzanilla cultivar resulted in mycorrhization, ranging from 1.90% for the AMF + CS30 treatment to 23.22% for the AMF treatment (Fig. 1a).

### Persistence of *M. brunneum* EAMa 01/58-Su strain in soil

In all treatments, an initial inoculum concentration of *Metarhizium* of 2.5 × 10^7^ CFU/g of soil was measured. In general, the persistence of the inoculum in the soil was higher in Picual cultivar treatments (*F*_(5,120)_ = 4.38; *P* = 0.0011) than in Manzanilla cultivar treatments (*F*_(5,120)_ = 1.67; *P* = 0.1477). The highest concentration was found in CS of Picual and Manzanilla cultivars with 7.58 × 10^5^ CFU/g of soil and 1.78 × 10^5^ CFU/g of soil, respectively (Fig. 1b). The amount of inoculum in the soil remained at levels between 10^4^ and 10^5^ CFU/g of soil for both cultivars. However, inoculum was not found in the soil of Manzanilla seedlings treated with AMF + MS (Fig. 1b).

### Fungal colonization of *M. brunneum* EAMa 01/58-Su strain in olive seedlings

None of the leaf samples showed colonization by the *M. brunneum* EAMa 01/58-Su strain in any of the soil treatments. Root colonization, however, was detected through both microbiological and molecular analyses (Table S2).

At 60 days, colonization was not observed using microbiological techniques but was quantified by qPCR and ddPCR. ddPCR allowed the detection of fungal DNA in treatments where qPCR was insufficient, such as CS, MS, AMF + CS, AMF + CS30, and AMF + MS30 in both cultivars. Fungal DNA in roots ranged from 0.17 ng/g root (CS, AMF + CS30, and AMF + MS30) to 0.48 ng/g root (MS) in Picual cultivar, and from 0.13 ng/g root (AMF + MS) to 0.62 ng/g root (CS) in Manzanilla cultivar (Table S2).

At 120 days, microbiological analysis revealed significant differences between treatments in Picual cultivar (*F*_(2,18)_ = 6.23; *P* = 0.008), but not in Manzanilla cultivar (F_(2,18)_ = 2.73; *P* = 0.09). Fungal growth on plates was observed only in root fragments from treatments containing conidia (CS, AMF + CS, and AMF + CS30) in both cultivars. The CS treatment showed the highest colonization percentage (21.43% in Picual cultivar and 11.43% in Manzanilla cultivar) compared with AMF + CS and AMF + CS30.

Molecular quantification confirmed colonization in treatments where microbiological detection was limited. For instance, qPCR measured fungal DNA in AMF + MS30 (Picual cultivar) and AMF + MS (Manzanilla cultivar), whereas ddPCR detected fungal DNA in MS, AMF + MS, and AMF + MS30 in Picual cultivar, and in MS and AMF + MS30 in Manzanilla cultivar (Table S2).

**Fig 2:**
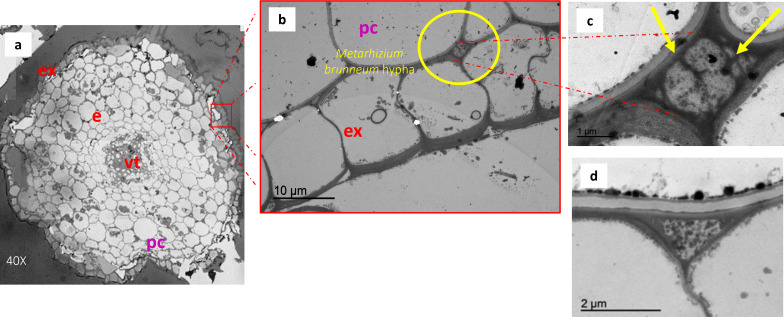
Cross-section of olive root from Picual cultivar treated with conidial suspension of *M. brunneum* EAMa 01/58-Su strain (CS treatment). (**a**) Transverse section of olive root 120 days after the CS treatment realized with optical microscopy image (40×). (**b, c**) TEM image of intercellular hypha between epidermis and cortex from treatment carried out with conidia. (**d**) TEM image of intercellular space in epidermis in control treatment. e, endodermis; ex, exodermis; pc, primary cortex; vt, vascular tissue.

Finally, colonization of the olive root cortex by *M. brunneum* EAMa 01/58-Su was observed using TEM, with the fungus located in the intercellular (apoplastic) space between the exodermis and the primary cortex (Fig. 2).

### Effect of the strain EAMa 01/58-Su on olive growth promotion

**Fig 3:**
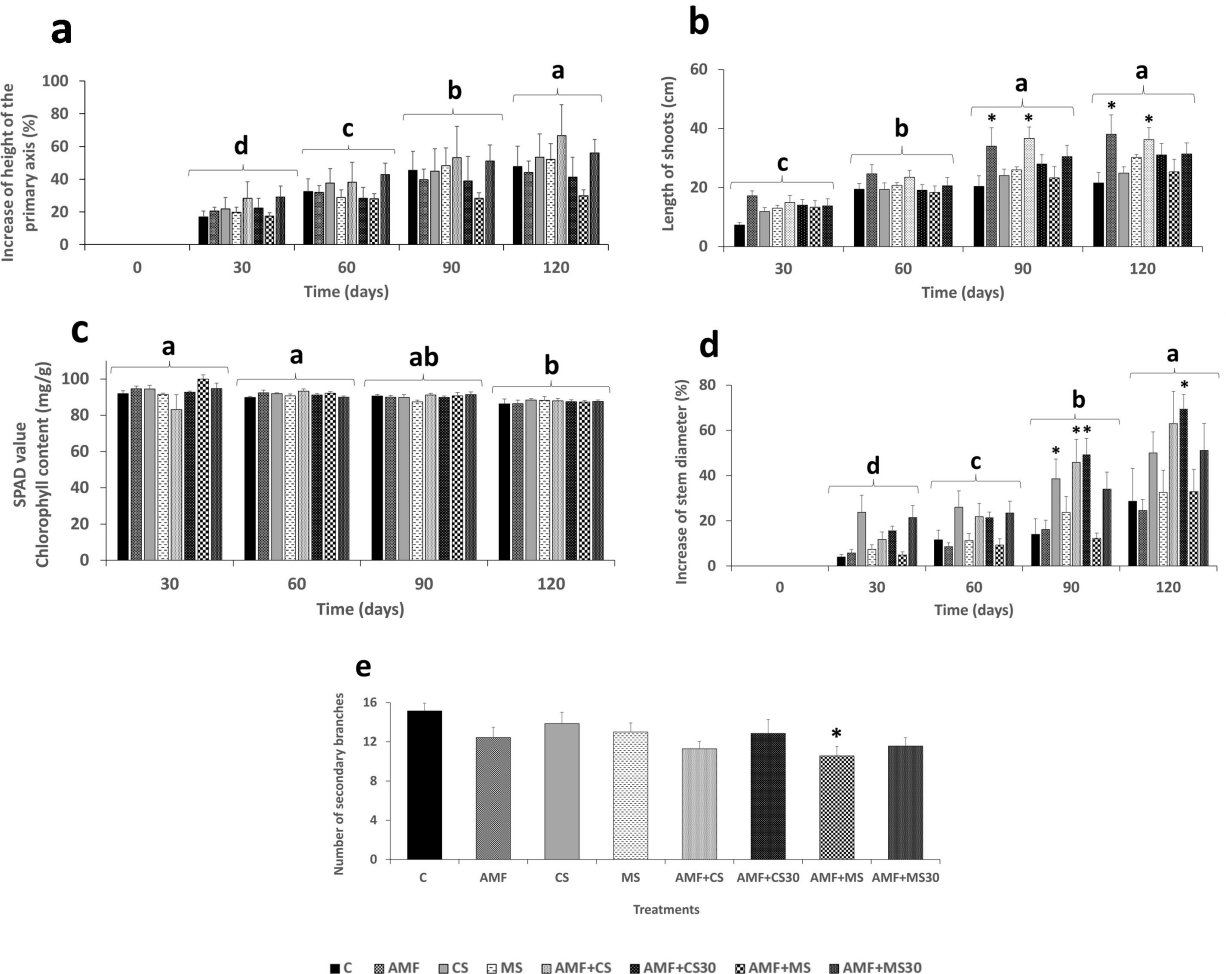
Growth parameters evaluated in the Picual cultivar. (**a**) Percentage increase in the height of the primary axis for each treatment at each evaluation time in the experiments, (**b**) shoot length (cm), (**c**) chlorophyll content (mg/g) (SPAD value), (**d**) percentage increase in stem diameter, and (**e**) number of secondary branches at the end of experiment. Error bars represent the standard error (SE) of the mean. Values thar do not share the same letter are significantly different at *P* ≤ 0.05 according to the Tukey-Kramer post hoc test (*α* = 0.05). Lowercase letters indicate comparisons between times, while asterisks indicate comparisons between treatments within each time point relative to the absolute control, according to the Dunnett’s test for multiple comparisons. Treatments: control (C); mycorrhiza (AMF); conidia (CS); microsclerotia (MS); mycorrhiza + conidia (AMF + CS); mycorrhiza + conidia applied 30 days after mycorrhiza application (AMF + CS30); mycorrhiza + microsclerotia (AMF + MS); and mycorrhiza + microsclerotia applied 30 days after mycorrhiza application (AMF + MS30).

**Fig 4:**
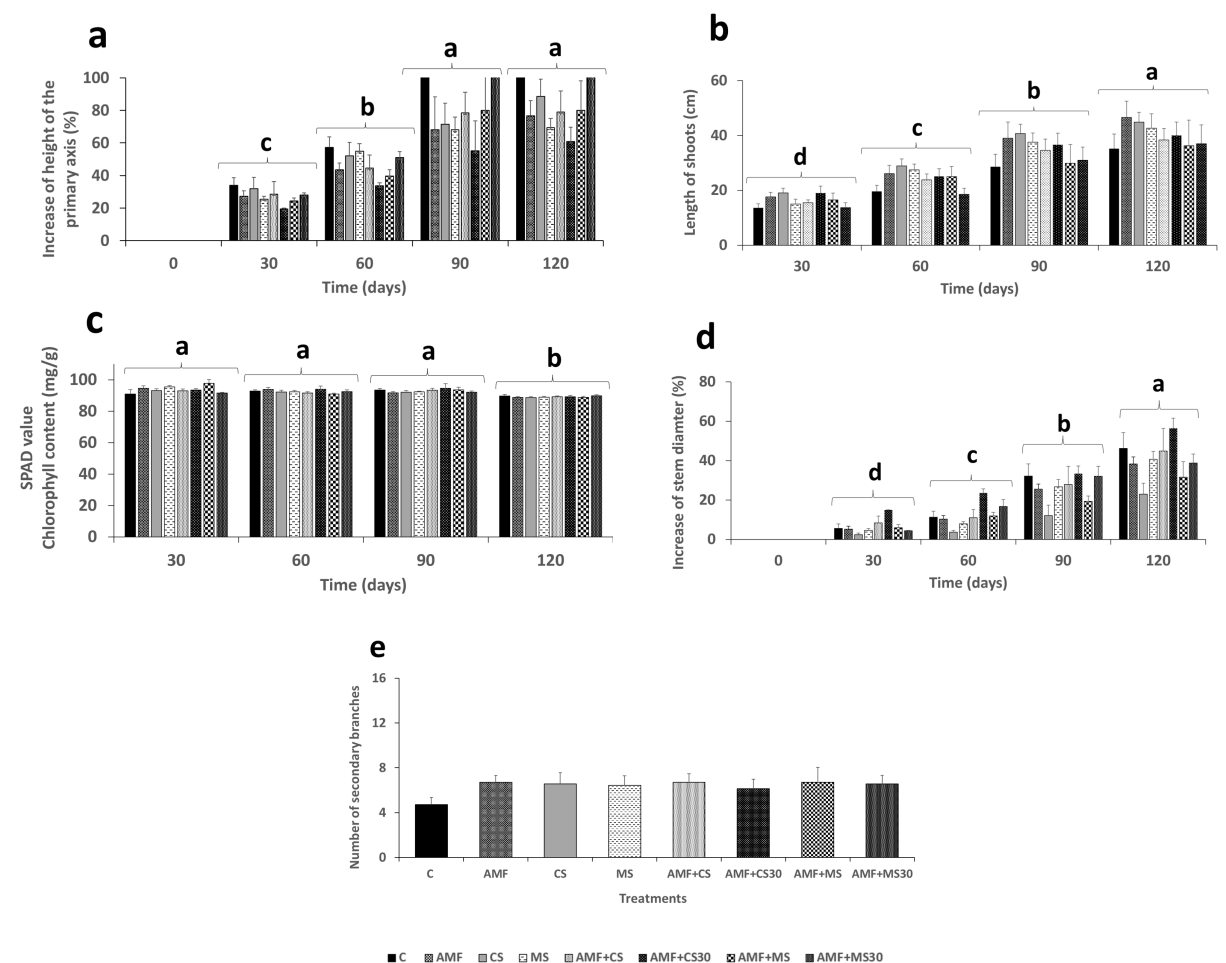
Growth parameters evaluated in the Manzanilla cultivar. (**a**) Percentage increase in the height of the primary axis for each treatment at each evaluation time in the experiments, (**b**) shoot length (cm), (**c**) chlorophyll content (mg/g) (SPAD value), (**d**) percentage increase in stem diameter, and (**e**) number of secondary branches at the end of experiment. Error bars represent the standard error (SE) of the mean. Values not sharing the same letter are significantly different at *P* ≤ 0.05 according to the Tukey-Kramer post hoc test (*α* = 0.05). Lowercase letters indicate comparisons between times, while asterisks indicate comparisons between treatments within each time point relative to the absolute control, according to the Dunnett’s test for multiple comparisons. Treatments: control (C); mycorrhiza (AMF); conidia (CS); microsclerotia (MS); mycorrhiza + conidia (AMF + CS); mycorrhiza + conidia applied 30 days after mycorrhiza application (AMF + CS30); mycorrhiza + microsclerotia (AMF + MS); and mycorrhiza + microsclerotia applied 30 days after mycorrhiza application (AMF + MS30).

Regarding the height of the primary axis, no significant differences were found between treatments within each cultivar or between cultivars. Differences were observed between time points within each cultivar (*F*_(3,31)_ = 84.95; *P* < 0.0001 in Picual [Table S3; Fig. 3a] and *F*_(3,31)_ = 61.31; *P* < 0.001 in Manzanilla [Table S3; Fig. 4a]). However, the length of shoots showed significant differences between treatments in the Picual cultivar. The AMF and AMF + CS treatments were significantly different and increased compared to the control at both 90 days (*F*_(7,31)_ = 4.21; *P* = 0.0006) and 120 days (*F*_(3,31)_ = 70.37 *P* < 0.001) (Fig. 3b). In the Manzanilla cultivar, there were only significant differences between times (*F*_(3,31)_ = 151.75; *P* < 0.001) (Fig. 4b).

The results showed significant differences in stem diameter between treatments at 90 and 120 days in both cultivars. In the Picual cultivar, the treatments CS, AMF + CS, and AMF + CS30 showed a significant increase compared to the control at 90 days, while only the treatment AMF + CS30 exhibited a significant increase at 120 days (*F*_(7,31)_ = 4.86; *P* < 0.0001) (Table S3; Fig. 3d). However, in the Manzanilla cultivar, only the CS treatment showed significant increase compared to the control at both 90 and 120 days (*F*_(7,31)_ = 4.56; *P* = 0.0003) (Table S3; Fig. 4d).

Regarding the number of secondary branches produced at the end of the experiment, significant differences were found between C and AMF + MS treatment in Picual (*F*_(7,48)_ = 2.13; *P* = 0.05) and ranged between 10.57 in AMF + MS and 15.14 in C treatments (Fig. 3e). However, no differences were found in the Manzanilla cultivar (F_(7,48)_ = 0.60; *P* = 0.75), ranging the number of secondary branches between 4.71 for C and 6.71 for each of the AMF, AMF + CS, AMF + MS treatments (Fig. 4e).

The SPAD chlorophyll index decreased over time and showed significant differences in both cultivars at the end of the experiment *F*_(3,31)_ = 8.06; *P* < 0.001 in Picual and *F*_(3,31)_ = 19.05; *P* < 0.0001 in Manzanilla (Fig. 3c and 4c) (Table S3).

**Fig 5:**
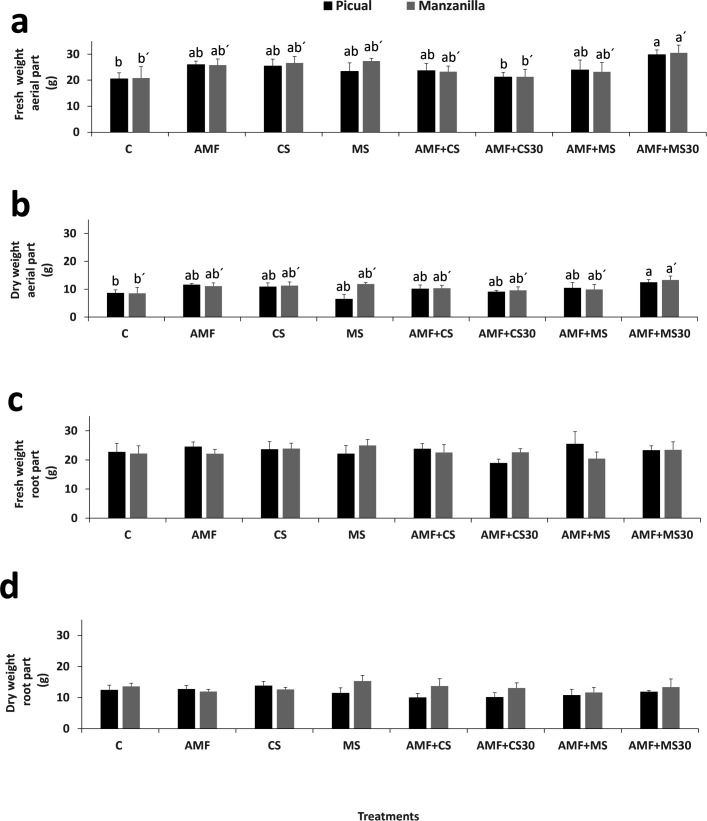
Fresh (**a**) and dry (**b**) weight of aerial part, fresh (**c**) and dry (**d**) weight of root part of olive plants in each one of the treatments for each cultivar (Picual and Manzanilla) at the end of the essay (120 days). Mean values (±SE) from seven plants in each instance followed by different letters are significantly different to one another according to the LSD test (*P* ≤ 0.05). Error bars indicate the standard error (SE) of the mean. Treatments: control (C); mycorrhiza (AMF); conidia (CS); microsclerotia (MS); mycorrhiza + conidia (AMF + CS); mycorrhiza + conidia applied 30 days after to applied mycorrhiza (AMF + CS30); mycorrhiza + microsclerotia (AMF + MS); and mycorrhiza + microsclerotia applied 30 days after to applied mycorrhiza (AMF + MS30).

Plant biomass was evaluated using both fresh and dry weights of the aerial and root parts. Significant differences between treatments were found for both fresh and dry weights, with the AMF + MS30 treatment differing from the control in both Picual and Manzanilla cultivars. Being *t*_48_ = 2.49; Prob = 0.0159 for fresh weight and *t*_48_ = 2.11; Prob = 0.0401 for dry weight in Picual cultivar and with *t*_48_ = 2.37; Prob = 0.0218 for fresh weight and *t*_48_ = 2.40; Prob = 0.0202 for dry weight for Manzanilla cultivars. Also, there were significant differences for fresh weight of the aerial part between the AMF + MS30 treatment and the AMF + CS30 in both cultivars, being *t*_48_ = 2.32; Prob = 0.0246 and *t*_48_ = 2.25; Prob = 0.0286 for Picual and Manzanilla, respectively (Fig. 5). In the root part, no significant differences were found between treatments for either fresh or dry weight (Fig. 5).

Finally, regarding the leaf nutrient profile, most macro- and micronutrient levels in both cultivars remained within ranges comparable to the control, although specific treatments induced significant shifts (Tables S4 and S5). In the Picual cultivar, the AMF + MS30 treatment significantly enhanced the nutritional status, with nitrogen (N) and potassium (K) levels increasing from low and medium ranges to the medium- and high-nutrition categories, respectively (Table S4).

In contrast, for the Manzanilla cultivar, the nutritional response was more homogeneous in the categories. While the control group started with high K levels, a significant decrease from the high to the medium range was observed in the CS treatment (Table S5). Additionally, treatments involving MS (MS and AMF + MS30) favored a higher accumulation of P, calcium (Ca), iron (Fe), zinc (Zn), and copper (Cu) compared to CS treatments (Table S5).

### Effect of the strain EAMa 01/58-Su on the expression of specific subsets of SR genes in olive

**Fig 6:**
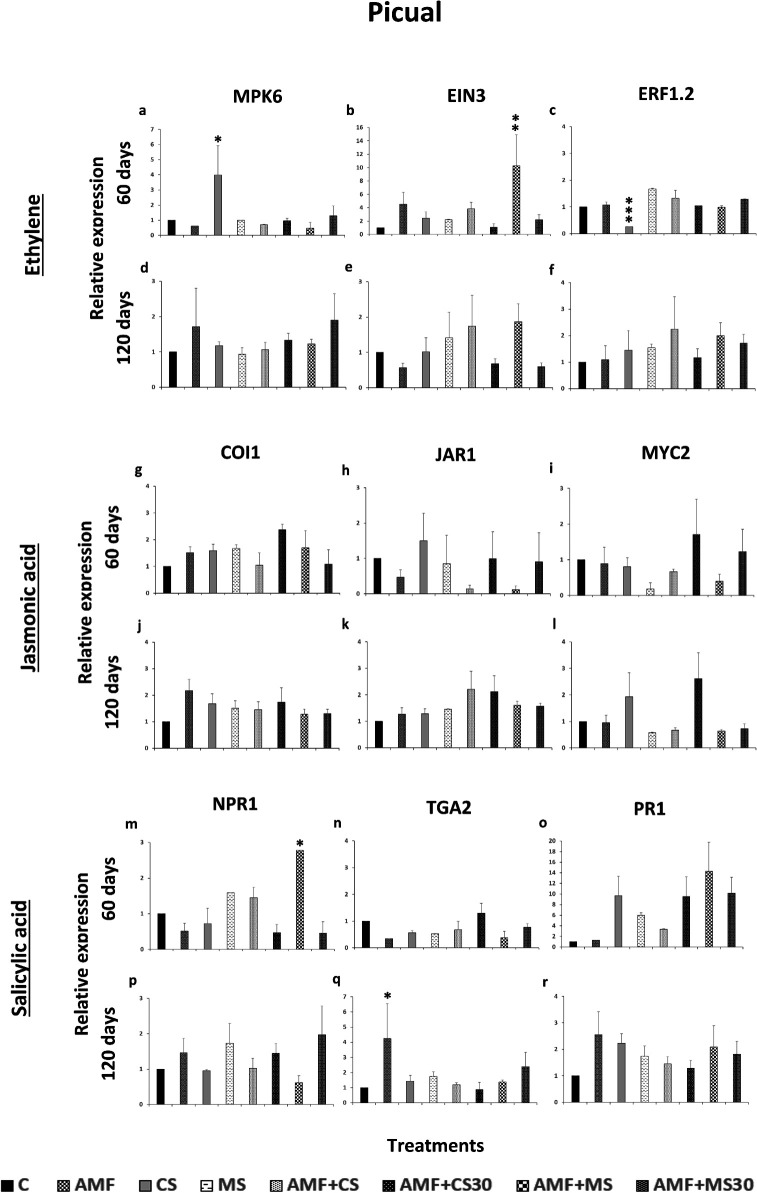
Expression patterns of MPK6, EIN3, and ERF1.2 of the ethylene pathway, of COI1, JAR1, and MYC2 of the jasmonic acid pathway and of NPR1, TGA2, and PR1 of the salicylic acid pathway in olive leaf cultivar Picual in response to colonization by *Metarhizium brunneum*. (**a–c**, **g–i**, **m–o**) In Picual cultivar’s treatments, 60 days after the beginning of the experiment. (**d–f**, **j–l**, **p–r**) In Picual cultivar’s treatments, 120 days after the beginning of the experiment. The data are averages of duplicate cDNA preparations, and vertical bars indicate the standard errors of the means resulting from 10 replicates from each treatment. The expression was detected by qRT-PCR and normalized to ACT1. Data of gene expression represent the mean of three or seven independent technical replicates for 60 or 120 days, respectively, according to the Dunnett’s test, * (*P* < 0.05), ** (*P* < 0.01), or *** (*P* < 0.001) over the bars indicate significant differences in relation to the control treatment. Treatments: control (C); mycorrhiza (AMF); conidia (CS); microsclerotia (MS); mycorrhiza + conidia (AMF + CS); mycorrhiza + conidia applied 30 days after applied mycorrhiza (AMF + CS30); mycorrhiza + microsclerotia (AMF + MS); and mycorrhiza + microsclerotia applied 30 days after applied mycorrhiza (AMF + MS30).

**Fig 7:**
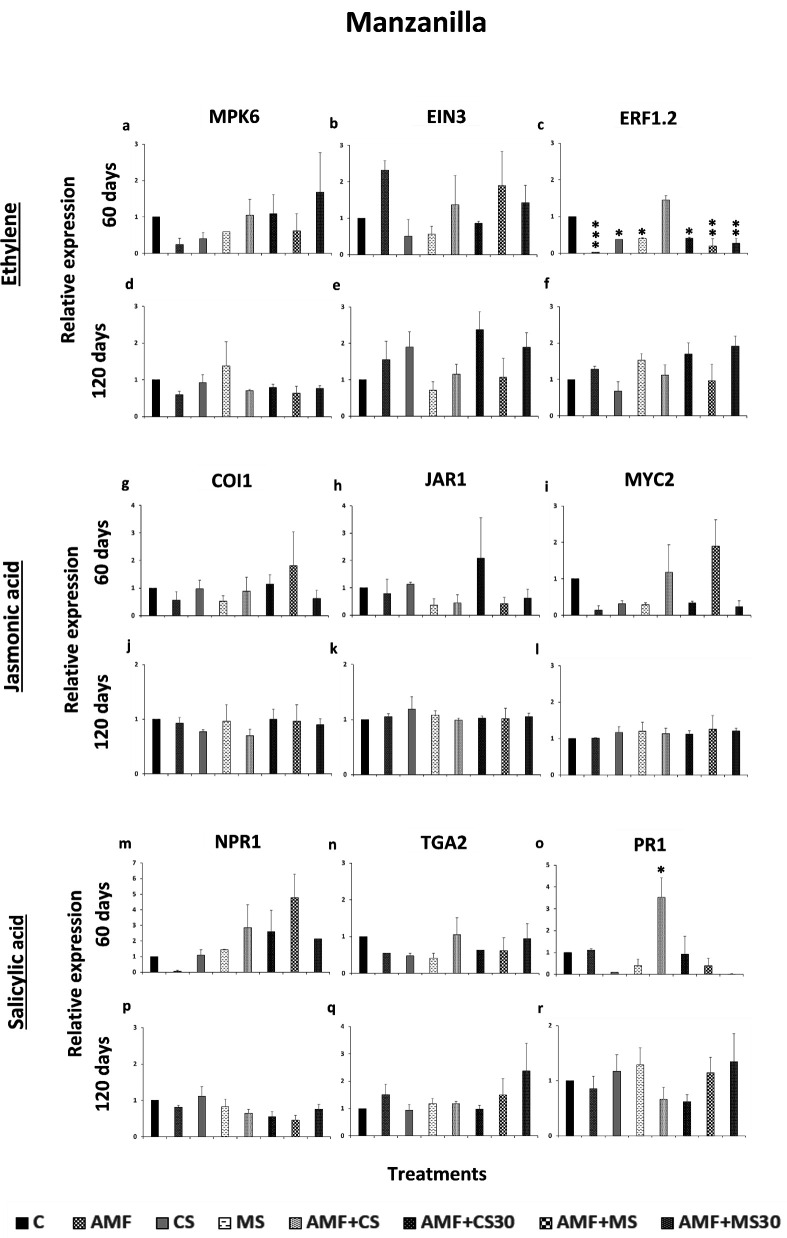
Expression patterns of MPK6, EIN3, and ERF1.2 of the ethylene pathway, of COI1, JAR1, and MYC2 of the jasmonic acid pathway and of NPR1, TGA2, and PR1 of the salicylic acid pathway in olive leaf cultivar Manzanilla in response to colonization by *Metarhizium brunneum*. (**a–c**, **g–i**, **m–o**) In Manzanilla cultivar’s treatments, 60 days after the beginning of the experiment. (**d–f**; **j–l**; **p–r**) In Manzanilla cultivar’s treatments, 120 days after the beginning of the experiment. The data are averages of duplicate cDNA preparations, and vertical bars indicate the standard errors of the means resulting from 10 replicates from each treatment. The expression was detected by qRT-PCR and normalized to ACT1. Data of gene expression represents the mean of three or seven independent technical replicates for 60 or 120 days, respectively, according to the Dunnett’s test, * (*P* < 0.05), ** (*P* < 0.01), or *** (*P* < 0.001) over the bars indicate significant differences in relation to the control treatment. Treatments: control (C); mycorrhiza (AMF); conidia (CS); microsclerotia (MS); mycorrhiza + conidia (AMF + CS); mycorrhiza + conidia applied 30 days after to applied mycorrhiza (AMF + CS30); mycorrhiza + microsclerotia (AMF + MS); and mycorrhiza + microsclerotia applied 30 days after to applied mycorrhiza (AMF + MS30).

Genes associated with induced SR (ISR) pathway were analyzed. In the ET biosynthesis pathway, the MPK6 and EIN3 genes were found to be upregulated in treatments that showed significant differences compared to the control, whereas the ERF1.2 gene was found to be downregulated (Fig. 6 and 7). Significant differences were found in both the Picual and Manzanilla cultivars at 60 days, but not at 120 days (Fig. 6a through c and 7c). In Picual, higher relative expression levels of MPK6 and EIN3 compared to the control were observed in CS and AMF + MS treatments, respectively (Fig. 6a and b). However, in the CS treatment, ERF1.2 showed a significant decrease in its relative expression compared to the control (Fig. 6c). Also, ERF1.2 showed a significant decrease in all the treatments except in AMF + CS treatment compared to the control in Manzanilla cultivar.

No differences in the relative expression of COI1, JAR1, and MYC2 in the JA biosynthesis pathway were detected in either cultivar at 60 or 120 days (Fig. 6g through l and 7g through l).

In the case of the three genes related to the SA pathway, NPR1 and TGA2 showed a significant increase in expression in the AMF + MS and AMF treatments compared to the control in the Picual cultivar at 60 and 120 days, respectively. Only PR1 showed a significant increase of its relative expression in the AMF + CS treatment compared to the control in the Manzanilla cultivar at 60 days. Neither NPR1 nor TGA2 showed a significant increase in their relative expression compared to the control in Manzanilla cultivar or at any time (Fig. 7).

## DISCUSSION

In recent years, interest in the soil microbiome has increased due to its effects on insect suppression and plant growth promotion (55). Among soil microbiota, endophytic entomopathogenic fungi (EEPF) have been studied for their ability to suppress insect pests (56–58) and pathogens (59, 60). Their interaction with the plant occurs in the phyllosphere, rhizosphere, and endosphere, modifying root structure, improving nutrient absorption, and by the plant, reducing the impact of abiotic and biotic stresses (61–65). Additionally, they promote plant growth by strengthening its defensive system against herbivores (66). However, the knowledge about all these beneficial interactions in woody plants is still very limited. The current work analyzes how different parameters influence growth and genetic responses during the colonization of olive roots by the *M. brunneum* EAMa 01/58- Su strain, a biocontrol agent against the olive fly *B. oleae,* and its interaction with other beneficial fungi such as AMF used in the propagation of olive plants by nurseries.

In this work, the presence of the EAMa 01/58-Su strain was detected in the roots of olive seedlings, but not in the leaves. Our results are consistent with previous studies showing that *Metarhizium* spp. applied to the soil is exclusively a root endophyte in several crops (37, 67, 68). The application of *Metarhizium* spp. CS is well-known as a successful inoculation method for many crops (32, 58, 69). However, to date, no studies have reported the endophytic colonization of plants after the application of MS. The microbiological, molecular, and histological techniques used in this study have, for the first time, shown the endophytic colonization of olive roots by *M. brunneum*. The ddPCR was shown to be more sensitive than qPCR and microbiological techniques, as it is much less affected by traces of contaminants and inhibitors that may remain in the roots after disinfection (70, 71). On the other hand, transmission images indicated that the colonization was restricted to the cortex, as observed with other fungi such as *Trichoderma harzianum* GFP22 used as biological control agents in olive trees (72). Wyrebek et al. (8) reported plant-rhizosphere-specific associations and indicated that *M. brunneum* was the species predominantly isolated from the roots of shrubs and woody crops, suggesting that it is likely rhizosphere-competent in these plants. In the rhizosphere, *M. brunneum* may compete with other beneficial fungi such as AMF, which form symbiotic associations with the roots of woody plants. This interaction may be driven by the induction of olive root exudates, which stimulate the germination of both AMF and the conidia or MS of *M. brunneum,* leading to the formation of infectious hyphae. These hyphae can either penetrate the olive roots and reach the vascular system in the case of AMF or remain confined in the cortex in the case of *M. brunneum*. These fungi can provide a range of benefits to the plant, including enhanced nutrient uptake, improved carbon and nitrogen exchange, and participation in N and P biochemical cycles (22, 73–76). Furthermore, olive cultivar plays a role in shaping the chemical composition of the exudates, which in turn affects the germination of *M. brunneum* propagules and its subsequent colonization. For instance, exudates from the Picual, Frantoio, and Arbequina cultivars differentially influence the germination of *Verticillium dahliae* conidia or MS (22, 73). These differences could explain the observed variation in *M. brunneum* colonization between the Picual and Manzanilla cultivars, as well as their respective interactions with AMF. It is even possible that root exudates from the Manzanilla cultivar exert a more fungistatic effect compared to those from Picual, potentially inhibiting *M. brunneum* colonization, thereby necessitating a higher inoculation dose of this EEPF in Manzanilla. Some studies confirm that genetic analyses carried out between Picual and Manzanilla present a high similarity, largely due to the great relationship existing within the same species (*O. europaea* L.) (77, 78) and the procedure of the Mediterranean basin, with approximately 86% genetic similarity in studies based on randomly amplified polymorphic DNA markers, they present moderate genetic differentiation according to diversity assessments based on expressed sequence-single nucleotide polymorphisms markers (79–81). This genetic divergence likely contributes to the observed differences in physiological responses, such as fruit characteristics, exudate composition, growth promotion, susceptibility to pests and diseases, adaptation to environmental conditions, and fungal colonization.

Our results also indicate that the Picual cultivar showed more differences in the growth parameters studied than the Manzanilla cultivar. This could be due to better retention of the inoculum throughout the entire experiment in the soil where the Picual cultivar was planted. However, it could also be explained, as is described by Santos-Rufo et al. (82) for the Picual and Frantoio cultivars, who demonstrated that the anatomical variations in the morphology of each cultivar can partly explain the differences in colonization and growth parameters between cultivars. Several studies have reported that EEPF can protect plants not only from phytophagous insects or pathogens, but also by altering root structure, thereby improving nutrient absorption (59, 60, 65). In our study, higher levels of nitrogen (N) and potassium (K) were measured in the AMF + MS30 treatment compared to the control in the Picual cultivar. In contrast, CS treatment had lower levels of K than the control in the Manzanilla cultivar. Differences in nutrient assimilation between the two cultivars may be due to their genotypes (83). Additionally, high levels of K are crucial for the proper growth, and its mobilization by endophytic microorganisms involved in the soil rhizophagy cycle is known to help host plants acquire nutrients (84–86).

The chlorophyll content of olive plants was not affected by EEPF colonization in either cultivar, as described by Ahmad et al. (87) and Greenfield et al. (88) in maize and cassava plants, respectively. These studies showed a neutral or negative effect of endophytic colonization on leaf chlorophyll content, contrary to (89), who reported an increase in soybean plants. Moreover, although the height of the main stem did not show differences between treatments in either cultivar, it was observed that, in general, the Picual cultivar had a greater stem diameter and higher number of secondary branches than the Manzanilla cultivar. This may be due to a higher percentage of AMF colonization in the Manzanilla compared to the Picual cultivar, which may lead to lower colonization by *M. brunneum*, as demonstrated by Zitlalpopoca-Hernandez et al. (90).

The present results, consistent with those obtained in maize plants, indicate that AMF may exert an antagonistic effect on the population density of EEPF when applied simultaneously, suggesting a possible competition for nutrients between both fungal groups associated with the roots and the mycelium produced by some AMF can suppress the development of other soil microorganisms (91, 92). Furthermore, a study reported that AMF can induce changes in the production of secondary metabolites or root exudates in plants such as *Medicago truncatula*, which may inhibit the growth and germination of EEPF, as observed in the case of *B. bassiana* (93). In addition, the cultivar itself may influence the quality and composition of root exudates, favoring AMF over EEPF colonization, as also suggested by Huang et al. (94).

In the case of the Picual cultivar, after 120 days, some treatments showed an inverse relationship between trunk diameter and the length of secondary branches when compared to the control, that is, a thinner trunk was associated with longer secondary branches, and vice versa. This phenomenon may be related to the concept of “Branching,” a plant strategy that reallocates carbon toward shoot development, thereby decreasing the wood-to-foliage ratio (95). This strategy may be of particular interest to nursery companies, as inoculating olive roots with *M. brunneum* could contribute to the development of a more robust aerial structure, ultimately enhancing transplant success and promoting fruiting capacity in the field. In this study, it was indicated that when both types of fungi were applied to the plant, they showed functional complementarity for plant protection and growth. However, the initial root inoculation by EEPF was reduced in the presence of AMF, as Zitlalpopoca-Hernandez et al. (90) demonstrates, while AMF was not affected by EEPF. On the other hand, the consortium between AMF and the EAMa 01/58-Su strain can be beneficial for the establishment of EEPF when applied after AMF. An increase in stem diameter and biomass has been observed when applied as CS and MS, respectively. The initial simultaneous application of AMF and EAMa 01/58-Su along with crop establishment may produce an antagonistic effect between them, influencing growth factors. These changes related to the stem diameter and biomass may be associated with modifications in root architecture, such as an increase in root hair density, lengths, and the emergence of lateral roots, thereby improving nutrient assimilation in plants colonized by *Metarhizium* spp. (19, 96).

Our results showed that inoculation with CS and MS of EAMa 01/58-Su strain can activate the ISR in olive, regardless of the colonization capacity. The EAMa 01/58-Su strain activated the ET and SA pathways, which are involved in ISR and acquired SR, respectively. The findings suggest a complex relationship between phytohormones, where the relative expression of ET and SA signaling genes showed variations at 60 days after the application of EAMa 01/58-Su strain to the soil. In this study, in the Picual cultivar, MPK6, signal receptor, and EIN3, a transcription factor in the ET pathway, activates a downstream cascade of gene expression, including the regulation of signaling genes such as ERF1.2 (97). In the olive tree, these genes were activated at 60 days, priming the plant for a rapid response to fungal colonization or abiotic stress. Regarding the SA pathway, TGA2 was activated at 120 days in the Picual cultivar, functioning as a transcription factor that ultimately triggers the plant’s defense responses.

In the case of the Manzanilla cultivar, at 60 days, only ERF1.2 of the ET pathway was activated, likely as a defense response to abiotic stress caused by temperature fluctuations in the greenhouse (Fig. S1), which were more pronounced in the treatments of this cultivar compared to Picual.

To conclude, this study reports for the first time that the EEPF *M. brunneum* EAMa 01/58-Su strain can colonize the root cortex of olive plants in two cultivars (Picual and Manzanilla). Nevertheless, since cultivar-specific traits such as root exudates may influence EEPF plant interactions, these findings should not be generalized to all olive cultivars. It should be noted, nonetheless, that the cultivars studied display sufficient genetic differentiation and represent varieties of major relevance in the olive oil sector. Further research, including a broader representation of olive genetic diversity, will be required to assess the general applicability of these results. However, differences arise when the plants have been previously mycorrhized. As a rhizosphere-competent fungus, it can promote plant growth and induce ISR during the colonization. The EAMa 01/58-Su strain, in addition to being an excellent mycoinsecticide against the olive fly, offers additional benefits as stimulation of growth and activation of the olive tree’s defense responses.

## SUPPLEMENTAL MATERIAL

Supplemental material aem.02048-25-s0001.docx Tables S1 to S5; Fig. S1.

ASM does not own the copyrights to Supplemental Material that may be linked to, or accessed through, an article. The authors have granted ASM a non-exclusive, world-wide license to publish the Supplemental Material files. Please contact the corresponding author directly for reuse.
