# Supplementary material for: Integration and benefits of root inoculation with endophytic entomopathogenic fungus Metarhizium brunneum in the propagation of olive tree seedlings in nurseries
Source: Appl Environ Microbiol. 2026 Jan 30;92(2):e02048-25. doi: 10.1128/aem.02048-25 (PMC12915339; doi:10.1128/aem.02048-25)
Supplement: Supplemental material — Tables S1 to S5; Fig. S1. [file aem.02048-25-s0001.docx]

**SUPPLEMENTAL MATERIAL**

| **TABLE S1** List of primers used for q-RT-PCR analysis | | | | | |
| --- | --- | --- | --- | --- | --- |
| Gene | Forward Sequence (5'-->3') | Reverse Sequence (5'-->3') | Product size | Rute | Function |
| MPK6 | ACGGTCGAAGATGCACTAGC | AAGGCGTCAAGCAGATAGGC | 79 | Ethylene | Signal receptor |
| EIN3 | TCATGGAAGTTTGCAGGGCT | GCAAGGCACTCAGCCTCATA | 166 | Ethylene | Transcription factor |
| ERF1/2 | GCCAAGCCATCCAAGTTGTT | CTTGAAGGGCTGCAAGTCTG | 134 | Ethylene | Response signal factor |
| COI1 | TGAGAATGACGGGGAATGGC | TCACCATGACCAGAGATGCG | 146 | JA | Signal receptor |
| JAR1 | GGGTTCCACAATTCGACTCC | CGCTTGCTTCGCCACTTATT | 229 | JA | JA activation |
| MYC2 | ATCTGGGTTCTTTCTGGCCC | GAAGATTTGTGCCGCTGACG | 83 | JA | Transcription factor |
| NPR1 | CAATCGCCTCTCAAACACCC | GCAACGGTGGACTGGAATTT | 124 | SA | Signal receptor |
| TGA2 | GCAACAGTTAGCACGGATCT | CAGCGCATCCATACCTTGTG | 82 | SA | Transcription factor |
| PR1 | CGAAAGGTATGCGGATTGCG | TTACTGCACGGAACCCTAGC | 231 | SA | Defense transcript |
| ACT1 | TTCACCACCTCAGCCGAAC | TGGCAGTCTCAAGATCCTGT | 94 | Housekeeping |  |

| **TABLE S2** Presence of *M. brunneum* EAMa 01/58-Su strain in olive roots at 60 and 120 days after soil treatment of two olive cultivars Picual and Manzanilla | | | | | | | |
| --- | --- | --- | --- | --- | --- | --- | --- |
|  | **Time** | **60 days** | | | **120 days** | | |
|  | **Technique** | **Microbiological** | **qPCR** | **ddPCR** | **Microbiological** | **qPCR** | **ddPCR** |
| **Treatments^1^** | **Cultivar** | **Colonization (%)** | **Amount** | **Amount** | **Colonization (%)** | **Amount of** | **Amount of** |
|  |  |  | **of DNAg** | **of DNAg** |  | **DNAg** | **DNAg** |
|  |  |  | **(ng/g root)^2^** | **(ng/g root)^2^** |  | **(ng/g root)^2^** | **(ng/g root)^2^** |
| **C** |  | 0 | 0 | 0 | 0 | 0 | 0 |
| **AMF** |  | 0 | 0 | 0 | 0 | 0 | 0 |
| **CS** |  | 0 | n.d. | 0.17 ± 1.76 x 10^-3^ a | 21.43 ± 7.99 a | n.d. | *** |
| **MS** | **Picual** | 0 | n.d. | 0.48 ± 0.02 a | 0 | n.d. | 0.34 ± 0.18 a |
| **AMF+CS** |  | 0 | n.d. | 0.23 ± 0.04 a | 5.71 ± 4.28 ab | n.d. | *** |
| **AMF+CS30** |  | 0 | n.d. | 0.17 ± 4.09 x 10^-3^ a | 2.86 ± 2.86 b | n.d. | *** |
| **AMF+MS** |  | 0 | 0.20 ± 0.02 | *** | 0 | n.d. | 0.23 ± 0.04 a |
| **AMF+MS30** |  | 0 | n.d. | 0.17 ± 2.09 x 10^-3^ a | 0 | (6.92 ± 1.25) x 10^-3^ | 0.18 ± 0.01 a |
| **C** |  | 0 | 0 | 0 | 0 | 0 | 0 |
| **AMF** |  | 0 | 0 | 0 | 0 | 0 | 0 |
| **CS** |  | 0 | n.d. | 0.62 ± 0.44 a | 11.43 ± 4.04 a | n.d. | *** |
| **MS** | **Manzanilla** | 0 | n.d. | 0.18 ± 0.01 a | 0 | n.d. | 0.17 ± 1.98 x 10^-3^ a |
| **AMF+CS** |  | 0 | n.d. | 0.19 ± 0.02 a | 1.43 ± 1.43 a | n.d. | *** |
| **AMF+CS30** |  | 0 | n.d. | 0.19 ± 0.01 a | 4.28 ± 2.97 a | n.d. | *** |
| **AMF+MS** |  | 0 | 0.13 ± 0.01 | *** | 0 | (8.13 ± 2.35) x 10^-3^ | *** |
| **AMF+MS30** |  | 0 | n.d. | 0.17 ± 0.12 x 10^-3^ a | 0 | n.d. | 0.17 ± 1.49 x 10^-3^ a |
| n.d.= not determined; total number of samples per treatment= 10; detection limit of DNA of the strain EAMa 01/58-Su: 3.1 x 10^-3^ ng/g of root.  ^1^Treatments: control (C); conidia (CS); microsclerotia (MS); mycorrhiza + conidia (AMF+CS); mycorrhiza + conidia applied 30 days after to applied mycorrhiza (AMF+CS30); mycorrhiza + microsclerotia (AMF+MS); mycorrhiza + microsclerotia applied 30 days after to applied mycorrhiza (AMF+MS30).  ^2^Same letters in the same column per cultivar indicate that there were no significant differences in the test of Tukey HDS (P≤0.05). ***: Samples sold out (this treatment has not been able to be processed by the ddPCR technique). | | | | | | | |

| **TABLE S3** Mixed model tests for fixed effects for the growth parameters: height of the primary axis, length of shoots, SPAD and stem diameter in the experiment over time. Significant factors are highlighted in bold | | | | | | |
| --- | --- | --- | --- | --- | --- | --- |
|  | **Picual** | | | **Manzanilla** | | |
|  | ***d.f.*** | ***F* value** | ***P* value** | ***d.f.*** | ***F* value** | ***P* value** |
| **Height of the primary axis** |  |  |  |  |  |  |
| Treatment | 7/31 | 0.73 | 0.6454 | 7/31 | 1.46 | 0.1944 |
| Time | 3/31 | 84.95 | **<0.0001** | 3/31 | 61.31 | **<0.0001** |
| Treatment x time | 21/31 | 1.20 | 0.2575 | 21/31 | 0.66 | 0.8647 |
| **Length of shoots** |  |  |  |  |  |  |
| Treatment | 7/31 | 4.21 | **0.0006** | 7/31 | 1.53 | 0.1702 |
| Time | 3/31 | 70.37 | **<0.0001** | 3/31 | 151.75 | **<0.0001** |
| Treatment x time | 21/31 | 0.80 | 0.7181 | 21/31 | 0.55 | 0.943 |
| **SPAD** |  |  |  |  |  |  |
| Treatment | 7/31 | 1.03 | 0.419 | 7/31 | 0.49 | 0.8408 |
| Time | 3/31 | 8.06 | **<0.0001** | 3/31 | 19.05 | **<0.0001** |
| Treatment x time | 21/31 | 1.50 | 0.081 | 21/31 | 1.41 | 0.1171 |
| **Stem diameter** |  |  |  |  |  |  |
| Treatment | 7/31 | 4.86 | **0.0001** | 7/31 | 4.56 | **0.0003** |
| Time | 3/31 | 104.34 | **<0.0001** | 3/31 | 229.50 | **<0.0001** |
| Treatment x time | 21/31 | 2.65 | **0.0003** | 21/31 | 1.95 | **0.0104** |
| **Treatments:** control (C); mycorrhiza (AMF); conidia (CS); microsclerotia (MS); mycorrhiza + conidia (AMF+CS); mycorrhiza + conidia applied 30 days after mycorrhiza application (AMF+CS30); mycorrhiza + microsclerotia (AMF+MS); mycorrhiza + microsclerotia applied 30 days after mycorrhiza application (AMF+MS30). **Times:** 30 days; 60 days; 90 days; 120 days. | | | | | | |

| **TABLE S4** Mean content and range of the main groups of elements present in leaf from seedlings of olive trees of Picual cultivar at the end of the experiment and results of ANOVA analysis (p-values of F test) to determine the effects of each element between treatments. For each treatment, mean values and standard error are shown | | | | | | | | | | | | | |
| --- | --- | --- | --- | --- | --- | --- | --- | --- | --- | --- | --- | --- | --- |
|  | **N**  **(% p/p)** | **P**  **(% p/p)** | **K**  **(% p/p)** | **Ca**  **(% p/p)** | **Mg**  **(% p/p)** | **Na (mg/kg)** | **Fe**  **(mg/kg)** | **Mn**  **(mg/Kg)** | **B**  **(mg/Kg)** | **Zn**  **(mg/Kg)** | **Cu (mg/Kg)** | **Ca + Mg + K** | **N + P + K** |
| **Control** | 1.27 ±  0.01 ab | 0.27 ±  0.00 a | 1.23 ± 0.03 ab | 0.89 ± 0.02 a | 0.10 ± 0.00 ab | 1130.38 ± 84.29 a | 76.00 ± 7.63 ab | 35.33 ± 1.45 a | 24.66 ± 1.20 a | 31.33 ±  0.66 a | 29.33 ± 6.00 a | 2.23 ±  0.03 ab | 2.80 ± 0.05 ab |
| **AMF** | 1.20 ±  0.03 bcd | 0.22 ±  0.01 b | 1.13 ± 0.02 bc | 0.80 ± 0.01 a | 0.09 ± 0.00 a | 1169.20 ± 28.71 a | 65.60 ± 2.37 ab | 32.46 ± 0.83 a | 22.80 ± 0.41 a | 23.66 ±  1.00 b | 21.26 ± 2.95 a | 2.04 ±  0.04 ab | 2.58 ± 0.06 bcd |
| **CS** | 1.21 ±  0.04 bcd | 0.21 ±  0.00 b | 1.10 ± 0.03 bc | 0.86 ± 0.02 a | 0.10 ± 0.00 ab | 1092.70 ± 4.66 a | 61.00 ± 3.51 b | 34.66 ± 2.40 a | 23.33 ± 0.66 a | 20.00 ±  1.15 c | 20.66 ± 6.76 a | 2.06 ±  0.03 bcd | 2.53 ± 0.03 cd |
| **MS** | 1.27 ±  0.03 abc | 0.28 ±  0.00 a | 1.15 ± 0.03 bc | 0.84 ± 0.06 a | 0.09 ± 0.00 ab | 1132.3 ± 25.12 a | 89.33 ± 11.72 a | 34.00 ± 3.05 a | 24.00 ± 1.00 a | 25.00 ±  0.57 b | 30.66 ± 8.19 a | 2.10 ±  0.05 abc | 2.70 ± 0.05 bc |
| **AMF+CS** | 1.23 ±  0.06 bcd | 0.18 ±  0.00 c | 1.08 ± 0.02 c | 0.75 ± 0.02 a | 0.09 ± 0.00 ab | 1203.00 ± 77.48 a | 65.33 ± 2.84 ab | 31.33 ± 2.40 a | 23.66 ± 0.33 a | 19.66 ±  0.66 c | 19.66 ± 4.97 a | 1.96 ±  0.03 cd | 2.53 ± 0.08 cd |
| **AMF+CS30** | 1.09 ±  0.00 d | 0.19 ±  0.00 c | 1.08 ± 0.00 c | 0.76 ± 0.04 a | 0.09 ± 0.00 ab | 1124.00 ± 53.46 a | 52.33 ± 1.85 b | 31.33 ± 2.40 a | 22.00 ± 1.00 a | 22.00 ±  1.00 bc | 14.33 ± 4.91 a | 1.93 ±  0.03 cd | 2.40 ± 0.00 d |
| **AMF+MS** | 1.10 ±  0.02 cd | 0.20 ±  0.00 bc | 1.05 ± 0.01 c | 0.77 ± 0.02 a | 0.09 ± 0.00 b | 1129.30 ± 63.22 a | 71.00 ± 3.78 ab | 33.66 ± 2.02 a | 21.66 ± 0.66 a | 22.00 ±  0.00 bc | 25.33 ± 5.36 a | 1.90 ±  0.00 d | 2.36 ± 0.03 d |
| **AMF+MS30** | 1.42 ±  0.02 a | 0.29 ±  0.00 a | 1.29 ± 0.03 a | 0.85 ± 0.02 a | 0.10 ± 0.00 ab | 1090.70 ± 30.38 a | 74.66 ± 3.75 ab | 31.66 ± 1.20 a | 24.00 ± 0.57 a | 30.33 ±  0.33 a | 20.33 ± 9.24 a | 2.26 ±  0.03 a | 3.03 ± 0.03 a |
| **Lower limit** | 1.40 | 0.09 | 0.65 | 1.00 | 0.13 | 100.00 | 30.00 | 25.00 | 19.00 | 10.00 | 6.00 | 2.00 | 1.80 |
| **Upper limit** | 2.00 | 0.15 | 1.20 | 2.50 | 0.30 | 800.00 | 150.00 | 90.00 | 75.00 | 80.00 | 120.00 | 3.50 | 3.50 |
| ***P*** | 0.00 | 0.00 | 0.00 | 0.05 | 0.05 | 0.18 | 0.01 | 0.78 | 0.31 | 0.00 | 0.74 | 0.00 | 0.00 |
| ***F* (7,23)** | 9.57 | 113.10 | 8.80 | 2.56 | 2.60 | 1.69 | 3.82 | 0.55 | 1.28 | 35.20 | 0.61 | 15.22 | 20.57 |
| Low nutrition | Normal nutrition | High nutrition |  | | | | | | | | | | |
|  |  |  |  |  |  |  |  |  |  |  |  |  |  |
| * The nutritional analysis was carried out with the Compositional Nutrient Diagnosis method (CND). Analysis carried out with 250 leaves per treatment with 10 olive seedlings per treatment. Means in the same column followed by the same letter are not significantly different according to the LSD test (P≤0.05). Treatments: control (C); mycorrhiza (AMF); conidia (CS); microsclerotia (MS); mycorrhiza + conidia (AMF+CS); mycorrhiza + conidia applied 30 days after to applied mycorrhiza (AMF+CS30); mycorrhiza + microsclerotia (AMF+MS); mycorrhiza + microsclerotia applied 30 days after to applied mycorrhiza (AMF+MS30) | | | | | | | | | | | | | |

| **TABLE S5** Mean content and range of the main groups of elements present in leaf from olive seedlings of Manzanilla cultivar at the end of the experiment and results of ANOVA analysis (p-values of F test) to determine the effects of each element between treatments. For each treatment, mean values and standard error are shown | | | | | | | | | | | | | |
| --- | --- | --- | --- | --- | --- | --- | --- | --- | --- | --- | --- | --- | --- |
|  | **N**  **(% p/p)** | **P**  **(% p/p)** | **K**  **(% p/p)** | **Ca**  **(% p/p)** | **Mg**  **(% p/p)** | **Na**  **(mg/kg)** | **Fe (mg/kg)** | **Mn (mg/Kg)** | **B (mg/Kg)** | **Zn (mg/Kg)** | **Cu**  **(mg/Kg)** | **Ca + Mg + K** | **N + P + K** |
| **Control** | 1.25 ±  0.06 a | 0.22 ±  0.00 ab | 1.37 ±  0.03 a | 0.92 ± 0.01 ab | 0.10 ± 0.00 a | 881.33 ± 3.75 b | 66.00 ± 3.00 a | 30.00 ± 1.15 a | 23.66 ± 0.33 a | 22.33 ± 0.33 c | 44.00 ± 5.03 a | 2.40 ±  0.05 a | 2.86 ± 0.03 a |
| **AMF** | 1.19 ±  0.04 a | 0.21 ±  0.00 ab | 1.28 ±  0.04 ab | 0.83 ± 0.02 b | 0.09 ± 0.00 a | 1084.33 ± 126.31 ab | 74.00 ± 5.03 a | 33.66 ± 1.45 a | 24.33 ± 0.33 a | 23.33 ± 1.20 bc | 52.00 ± 3.21 a | 2.20 ±  0.10 a | 2.70 ± 0.00 ab |
| **CS** | 1.12 ±  0.05 a | 0.20 ±  0.00 b | 1.20 ±  0.03 b | 0.83 ± 0.00 b | 0.08 ± 0.00 b | 1193.66 ± 82.79 a | 63.66 ± 2.66 a | 30.66 ± 0.33 a | 22.66 ± 0.33 a | 22.00 ± 0.57 c | 41.66 ± 0.66 a | 2.13 ±  0.03 a | 2.56 ± 0.06 b |
| **MS** | 1.26 ±  0.02 a | 0.26 ± 0.01 a | 1.24 ±  0.00 ab | 0.93 ± 0.63 ab | 0.09 ± 0.00 ab | 859.33 ± 34.10 b | 76.00 ± 4.04 a | 33.33 ± 1.33 a | 23.66 ± 0.33 a | 26.66 ± 0.66 a | 51.33 ± 8.41 a | 2.26 ±  0.06 a | 2.76 ± 0.03 ab |
| **AMF+CS** | 1.16 ±  0.02 a | 0.21 ±  0.00 ab | 1.28 ±  0.03 ab | 0.91 ± 0.01 ab | 0.09 ± 0.00 ab | 994.66 ± 55.85 ab | 69.66 ± 2.40 a | 35.66 ± 0.88 a | 24.33 ± 1.45 a | 22.33 ± 0.33 c | 47.66 ± 2.96 a | 2.26 ±  0.03 a | 2.70 ± 0.00 ab |
| **AMF+CS30** | 1.16 ±  0.02 a | 0.24 ±  0.00 ab | 1.27 ±  0.01 ab | 0.89 ± 0.02 ab | 0.08 ± 0.00 b | 1125.00 ± 49.21 ab | 69.33 ± 4.63 a | 35.00 ± 0.57 a | 22.33 ± 0.66 a | 22.33 ± 0.33 c | 43.66 ± 3.75 a | 2.26 ±  0.03 a | 2.70 ± 0.00 ab |
| **AMF+MS** | 1.23 ±  0.04 a | 0.25 ±  0.02 ab | 1.31 ±  0.04 ab | 0.95 ± 0.03 a | 0.10 ± 0.00 a | 995.00 ± 31.66 ab | 73.33 ± 4.37 a | 31.33 ± 0.66 a | 24.33 ± 0.88 a | 26.00 ± 0.57ab | 51.00 ± 8.62 a | 2.36 ±  0.06 a | 2.80 ± 0.05 a |
| **AMF+MS30** | 1.23 ±  0.06 a | 0.25 ±  0.01 ab | 1.33 ±  0.00 ab | 0.96 ± 0.01 a | 0.10 ± 0.00 a | 923.33 ± 16.19 ab | 77.00 ± 8.88 a | 35.66 ± 2.60 a | 21.33 ± 1.20 a | 23.00 ± 0.57 bc | 55.33 ± 13.92 a | 2.40 ±  0.06 a | 2.80 ± 0.05 a |
| **Lower limit** | 1.4 | 0.09 | 0.65 | 1.00 | 0.13 | 100.00 | 30.00 | 25.00 | 19.00 | 10.00 | 6.00 | 2.00 | 1.80 |
| **Upper limit** | 2.00 | 0.15 | 1.20 | 2.50 | 0.00 | 800.00 | 150.00 | 90.00 | 75.00 | 80.00 | 120.00 | 3.50 | 3.50 |
| ***P*** | 0.40 | 0.01 | 0.03 | 0.00 | 0.00 | 0.01 | 0.47 | 0.05 | 0.13 | 0.00 | 0.84 | 0.37 | 0.00 |
| ***F* (7,23)** | 1.11 | 3.87 | 2.87 | 4.68 | 6.13 | 3.7 | 0.99 | 2.99 | 1.90 | 8.12 | 0.47 | 2.88 | 5.04 |
| Low nutrition | Normal nutrition | High nutrition |  | | | | | | | | | | |
|  |  |  |  |  |  |  |  |  |  |  |  |  |  |

* The nutritional analysis was carried out with the Compositional Nutrient Diagnosis method (CND). Analysis carried out with 250 leaves per treatment with 10 olive seedlings per treatment. Means in the same column followed by the same letter are not significantly different according to the LSD test (P≤0.05). Treatments: control (C); mycorrhiza (AMF); conidia (CS); microsclerotia (MS); mycorrhiza + conidia (AMF+CS); mycorrhiza + conidia applied 30 days after to applied mycorrhiza (AMF+CS30); mycorrhiza + microsclerotia (AMF+MS); mycorrhiza + microsclerotia applied 30 days after to applied mycorrhiza (AMF+MS30)


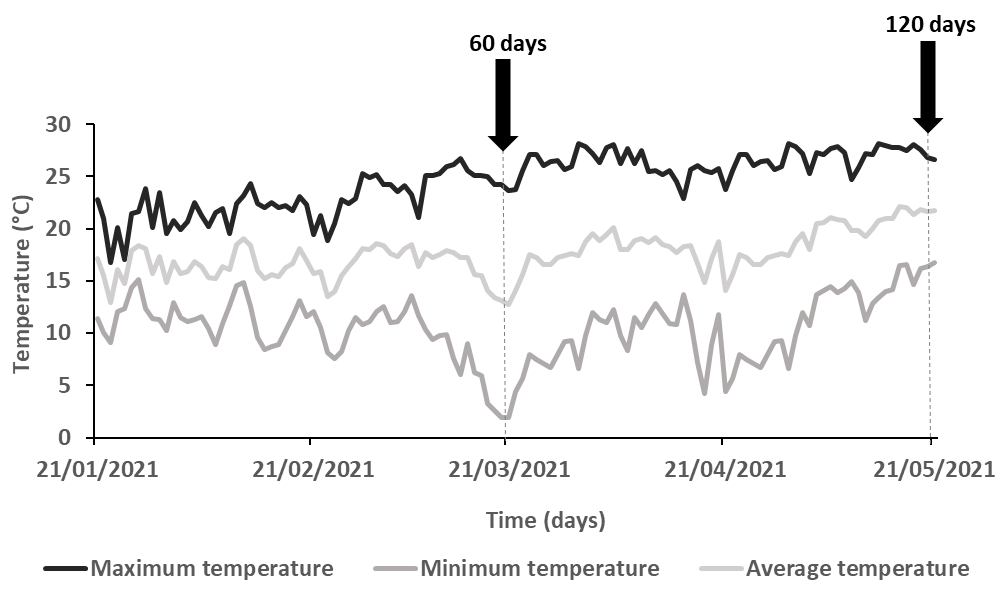


**FIG S1** Daily temperatures recorded throughout the entire evaluation period of the greenhouse experiment. Measurements carried out with Data Logger TGP-4520 (BERMAN Termómetros e Instrumentación S.L (Barcelona, Spain)
